# Management of high-output cardiac failure caused by head and neck AVMs: Percutaneous suture-assisted ethanol and coil embolotherapy

**DOI:** 10.3389/fcvm.2022.949558

**Published:** 2022-10-26

**Authors:** Ming-zhe Wen, Xin-yu Li, Yu-chen Shen, Zhen-feng Wang, Lian-zhou Zheng, De-ming Wang, Xin-dong Fan, Li-xin Su, Xi-tao Yang

**Affiliations:** ^1^Department of Interventional Therapy, Multidisciplinary Team of Vascular Anomalies, Shanghai Ninth People's Hospital, Shanghai JiaoTong University School of Medicine, Shanghai, China; ^2^Department of Neurosurgery, Shanghai Ninth People's Hospital, Shanghai JiaoTong University School of Medicine, Shanghai, China

**Keywords:** high output cardiac failure, ethanol, coils, embolization, arteriovenous malformations

## Abstract

**Purpose:**

The aim of this study was to describe the treatment technique, outcomes, and complications of Schobinger stage IV head and neck arteriovenous malformations (HNAVMs) with associated high-output cardiac failure (HOCF) using ethanol and coils with the percutaneous suture technique.

**Methods:**

From January 2015 to December 2019, 19 patients who had HNAVMs with associated HOCF were treated first with a percutaneous suture of the remarkably dilated dominant drainage vein (RDDOV) and subsequent embolization with coils and ethanol. The percutaneous suture of RDDOV was preferred to be performed, followed by the deployment of coils and the injection of absolute ethanol *via* transarterial approach, direct puncture approach, or both of them. Treatment outcomes and complication rates were evaluated at follow-up.

**Results:**

A total of 19 patients who experienced HNAVMs with HOCF received 19 percutaneous suture procedures and 84 embolization procedures with ethanol and coils. Complete or >90% shunt reduction of the AVM was achieved in 16 patients. Notably, 19 patients with New York Heart Association (NYHA) stage II improved to stage I, and the symptom of dyspnea disappeared after embolization. The symptoms of five patients with bleeding disappeared. All patients presented with cosmetic concerns; Four were cured, and eight had a clearly recognizable improvement. Of note, 19 (100%) patients presented with impairment of daily life, which was resolved. The minor complications were encountered and recovered by the self. No major complications occurred.

**Conclusion:**

This study provides evidence that ethanol and coil embolotherapy is effective and safe in treating HOCF caused by HNAVMs with acceptable complications in these cases. The percutaneous suture technique for RDDOV management can act as an adjunct for embolotherapy.

## Introduction

Head and neck arteriovenous malformations (HNAVMs) are rare vascular lesions characterized by high-flow shunts bypassing the intervening capillary bed (1). The HNAVMs could progress and aggress, causing aesthetic and/or functional complications, such as intolerable pulsatile swelling, hemorrhage, ulceration, or life-threatening high-output cardiac failure (HOCF) as Schobinger stage IV lesions (2). This life-threatening situation warrants an early and prompt medical intervention.

Due to the rarity of HOCF induced by HNAVMs, appropriate treatment remains confusing. Aggressive surgical resection makes the lesion worse by causing growth and recruitment of adjacent tissue (3). Palliative management, including the ligation or proximal embolization of feeding arteries, plays a limited role in temporarily relieving symptoms. However, a long-term follow-up showed poor clinical and imaging outcomes, including recurrence and exacerbation (4). Management of HOCF with HNAVMs is treated with the closure of excessive shunts. Endovascular embolization has evolved as a preferred alternative, especially with ethanol given the decrease in recurrence rate, and the possibility of cure, without disfigurement and functional sacrifice of the cranial nerves (5), but less is published on embolotherapy for HNAVMs in Schobinger stage IV.

At present, there is a paucity of consensus on a standardized treatment strategy for HOCF caused by HNAVMs due to their rarity. The aim of the study was to retrospectively analyze the technical and clinical safety and the effectiveness of the ethanol and coil embolization of HNAVMs with HOCF with the percutaneous suture technique.

## Methods

### Patients

The Institutional Review Board approved a review and utilization of patient medical and imaging records with a waiver of informed consent from patients given its retrospective nature. As tertiary centers specializing in peripheral AVMs, our institutions draw patients from across China.

A total of 19 patients from the electronic medical record system (nine women; 10 men; mean age, 29 years; age range, 18–47 years) with HOCF caused by HNAVMs were admitted to our institution and received percutaneous suture-assisted ethanol and coil embolization between January 2015 and December 2019. Patients were eligible if (i) their HOCF caused by HNAVMs was confirmed by color Doppler ultrasound and New York Heart Association (NYHA) class stage II; (ii) their signs and symptoms of HOCF were suggestive of Schobinger grade IV HNAVMs, which were confirmed by physical examination, contrasted computed tomography, and digital subtraction angiography (DSA) performed during endovascular treatment; and (iii) the location of the lesions was located in the head and neck.

Exclusion criteria included a history of hypertension, diabetes mellitus, valvular heart disease, coronary artery disease, and a contraindication for ethanol. A total of 6 AVMs in other parts of the body were excluded from the analysis during that time frame.

The most common presenting symptoms were intolerable pulsation, dizziness, and dyspnea on exertion, occurring in all patients (100%). Baseline demographic data are shown in Table 1. According to the NYHA classification of heart failure, 13 patients were in NYHA II, five patients in NYHA III, and 1 patient in NYHA IV. Other symptoms included expansion of redness (*n* = 6, 31.6%) and bleeding (*n* = 3, 15.8%). A total of 5 patients used anti-failure medication (sacubitril and valsartan sodium tablets and furosemide tablets). All patients had a high psychosocial burden when they presented to our department.

**Table 1:** Baseline demographic information.

**Patient no.sex/age,year**	**Previous Treatment**	**Indication**	**NYHA classification**	**Location**	**No. of suture procedures**	**No. of sessions**	**Embolic Agent**			**Complications**	**Follow-up period, months**	**Schobinger classification post embolization**	**Degree of devascularization (%)**	**Outcome**
							**Number of coils**		**Volume of Ethanol in Each Procedure (mL)**					
							**Detachable Coils**	**Undetachable Coils**						
1/M/19	Feeding artery ligation	HOCF, P,S,W,V	II	Right, MR	2	2	8	149	45/28	Facial numbness	7	I	>90%	Major improvement
2/M/29	Nil	HOCF, P,S,W,V	III	Right, MR	2	5	14	128	30/34/27/25/5	None	15	I	>90%	Major improvement
3/M/37	Feeding artery embolization	HOCF, P,S,W,V	III	Right, MR	2	4	5	62	45/40/37/10	None	5	I	>90%	Major improvement
4/F/32	Surgical resection	HOCF, P,S,W,V,B,R	II	Right, MR	2	6	17	243	50/50/45/38/36/27	Blister, Suture-associated infection	20	II	>75%	Minor improvement
5/F/47	Nil	HOCF, P,S,W,V	II	Right, MR	1	3	19	237	50/50/25	None	36	I	>90%	Minor improvement
6/F/41	Nil	HOCF, P,S,W,V	II	Right, MR	2	2	11	164	45/40	Facial numbness,Blister	4	II	100	C complete relief
7/F/32	Nil	HOCF, P,S,W,V,E,R	II	Right, MR	2	4	8	84	35/15/10/8	Blister	3	I	>90%	Major improvement
8/M/25	Nil	HOCF, P,S,W,V	II	Right, MR	3	4	26	282	40/45/50/35	None	34	I	>90%	Major improvement
9/F/25	Feeding artery embolization	HOCF, P,S,W,V,E, R	IV	Left, MR	3	8	22	457	40/33/52/45/45/30/45/35	Facial numbness, Blister, Suture-associated infection	12	II	>75%	Minor improvement
10/M/18	Nil	HOCF, P,S,W,V	II	Left, MR	3	4	17	141	20/20/10/5	None	8	I	100	C complete relief
11/F/39	Nil	HOCF, P,S,W,V, U,B	III	Right, MR	3	6	10	167	40/36/40/35/38/20	Blister	9	II	>75%	Minor improvement
12/M/29	Feeding artery embolization	HOCF, P,S,W,V	II	Right, MR	2	3	24	237	35/30/20	Erosion and infection of coils	13	I	100	C complete relief
13/F/26	Nil	HOCF, P,S,W,V, U	II	Left, MR	2	4	6	142	45/41/28/40	Blister	22	I	>90%	Minor improvement
14/M/20	Nil	HOCF, P,S,W,V	II	Left, MR	2	3	35	103	27/15/12	None	3	I	100	C complete relief
15/F/32	Nil	HOCF, P,S,W,V,U,B	III	Right, MR	2	7	9	165	43/45/50/48/45/20/5	Blister, Erosion and infection of coils	33	I	100	Major improvement
16/M/35	Nil	HOCF, P,S,W,V,U,B	II	Left, MR	2	4	10	145	45/33/30/15	Blister	40	I	>90%	Minor improvement
17/F/28	Nil	HOCF, P,S,W,V	II	Right, MR	2	4	4	147	39/50/50/30	None	38	I	>90%	Major improvement
18/M/22	Nil	HOCF, P,S,W,V,U	III	Right, MR	3	5	29	276	30/50/35/24/20	Erosion and infection of coils	4	I	>90%	Major improvement
19/M/44	Feeding artery embolization	HOCF, P,S,W,V,U,B	II	Right, MR	2	6	7	186	40/50/43/39/33/14	None	15	I	>90%	Minor improvement

B, bleeding; HOCF, high-output cardiac failure; MR, maxillofacial region; P, pulsation; R, redness; S, swelling; U, ulceration; V, vasodilation; W, warmth.

Of 19 patients, 6 (31.6%) had undergone unsuccessful treatment in another hospital before admission to our institution. Of those 6 patients, 4 (20%) had feeding artery embolization, 1 (5%) had feeding artery ligation, and 1 (25%) had an unsuccessful resection and recurrent lesions that worsened and progressed. A total of 19 patients reported the presence of HNAVM at birth, and 6 (31.6%) noted the recognizable expansion of the lesion during pregnancy (*n* = 4) or injury (*n* = 2).

On the first visit to the Vascular Malformation Center, the establishment of the treatment strategy was performed based on the multidisciplinary approach. A decision on the planned treatment method was based on a combined discussion among interventional radiologists, anesthesiologists, cardiologists, and respiratory and vascular surgeons. Other combined problems, including skin necrosis, nerve palsy due to longstanding arteriovenous shunting, and cosmetic deformity, were treated through a multidisciplinary team approach, including a psychiatrist, neurologist, dermatologist, and plastic surgery specialist.

### Techniques

All embolization procedures were performed under general anesthesia. The continuous monitoring of pulmonary artery pressure was performed using the Swan-Ganz catheter in case of the expected total ethanol dosage exceeded 0.5 ml/kg. Baseline angiography was used to assess the extent and hemodynamic characteristics of AVM *via* femoral arterial access. The angiographic findings determined the choice of treatment methods. The angiographic classification was based on the consensus of two interventional radiologists according to the classification system reported by Yakes classification (6) to make treatment plans for AVMs.

**Figure 1:**
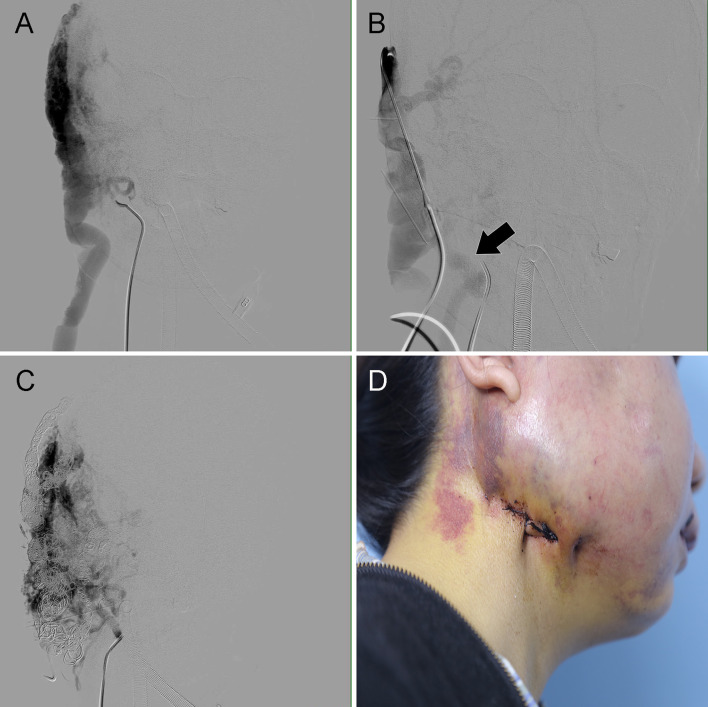
Case 4 with the complaint of exertional palpitation and progressive breathlessness for 1 year. An HOCF patient with right neck arteriovenous malformations (AVMs). **(A)** Anteroposterior angiogram shows the location of the dilated RDDOV in the venous phase. **(B)** Venography through an 18-gauge needle shows the RDDOV becoming narrow after the RDDOV was sutured with a 3-0 absorbable suture (black arrow). **(C)** Anteroposterior angiogram shows that the RDDOV was embolized by the coils. HOCF, high-output heart failure. There is still residual AVM that would be considered in the >99% occlusion category. **(D)** Photograph shows the location of the RDDOV suture.

**Figure 2:**
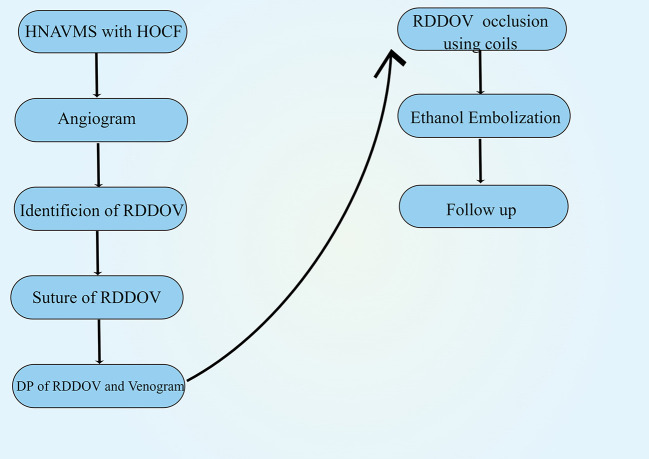
Schematic depiction of the treatment of head and neck arteriovenous malformations with associated high-output cardiac failure. HNAVMs = head and neck arteriovenous malformations; HOCF, high-output cardiac failure; RDDOV, remarkably dilated dominant drainage vein.

The remarkably dilated dominant drainage vein (RDDOV) was identified on the angiogram (Figure 1A) and color Doppler ultrasonography. The location of the suture is defined as the zone of the nidus as close as possible to the outlet of RDDOV (Figure 1B). The needle entrance and exit depended on the size of the RDDOV. The intended RDDOV was tied on the skin using a 3-0 absorbable suture with the assistance of external compression (Figure 1D). The needle was passed through the vein under the guidance of color Doppler ultrasonography. The success of the suture is defined as >75% venous outflow stenosis owing to the difficulty of closing the vein completely with the suture. After the confirmation of the slow flow in the AVM lesions, ethanol and coil embolotherapy was initiated through a transarterial or percutaneous puncture needle. Transarterial embolization is the preferred method. In case of the failure of transarterial embolization, a direct puncture approach was performed. A 16-gauge needle (Cook, Bloomington, IN, USA) percutaneously punctured the RDDOV under guidance (Figure 1B). A 2.2 F microcatheter (Asahi, Seto, Japan) was guided through the needle into the dilated venous sac and the tip of the microcatheter was placed as close as possible to the outlet of RDDOV. After confirming the correct position of the microcatheter, RDDVO was first embolized with the three-dimensional coil (TDC) (Acheva, Shanghai, China), followed by a Nester embolization coil (Cook, Bloomington, IN) through the microcatheter. Before coil deployment, the coil configuration under fluoroscopy was observed to assess any changes that might indicate instability and the risk of migration. Following satisfactory placement and observation for stability, the coils were detached one by one in the usual fashion until the identification of residual flow was confirmed by venography (Figure 1C). Absolute ethanol was injected as if the microcatheter was being pulled back. Typically, there was no flow in previously reported arterial vessels feeding the AVM after embolization. When residual flow was identified in repeat angiography from the arterial catheter, multiple percutaneous punctures with 21G butterfly needles with ethanol were appropriate for sclerotherapy under fluoroscopic guidance. The appropriate volume and the rate of bolus ethanol injection amount depended on the manual injection contrast medium that fills the AVM nidus and avoids over-injection with reflux into small feeding arteries. The technique of treating HNAVMs with HOCF is summarized in Figure 2.

In most cases, absolute ethanol (99.7%) was used, but superficially located AVMs were embolized with diluted (50%) ethanol with contrast medium. After the embolization procedure, completion angiography was used to assess that the nidus was completely devascularized.

The use of ethanol was limited to 1 ml/kg as the total amount and 0.1 ml/kg body weight as the maximum single-injection volume. After the injection of ethanol, the next injection was performed for 5–10 min to evaluate shunt closure and changes in flow dynamics. After ethanol embolization, management included intravenous infusion of fluids and dexamethasone. Hemoglobinuria observed during ethanol embolization was treated by hydration with intravenously administered lactated Ringer solution (2,000 ml).

The treatment interval between the treatment sessions was 4 weeks on average. The endpoint of embolization treatment was refined as the absence of HOCF, free from clinical discomfort, and >90% disappearance of the AVM on angiography. However, for certain AVMs patients, such as those that were easy to achieve complete embolization, reaching the absence of clinical discomfort with the complete disappearance of residual AVM was thought to complete the treatment program.

### Follow-up

Follow-up was completed in the outpatient clinic with physical examination, color Doppler ultrasound, and Quality of Life (QoL) questionnaire at 3-month intervals. DSA was performed under local anesthesia at 3- and 12-month intervals after the final treatment and annually after the first DSA follow-up. The radiologic follow-up period ranged from 3 to 40 months (mean, 16.8 months). Additional therapies were recommended in cases of reappearance of AVM nidus or symptoms at follow-up. Pretreatment and posttreatment DSA were assessed by two experienced interventional neuroradiologists in consensus. The DSA results after treatment were classified into five grades, namely, (1) <50% shunt reduction; (2) >50% and <75% shunt reduction; (3) >75% shunt reduction; (4) >90% shunt reduction; and (5) complete occlusion of the AVM. The patients' symptoms and QoL were evaluated using QoL according to the previous report (7). This included symptoms such as bleeding, cosmetic deformity, cardiac functional impairment, and daily life impairment. The comparison of their symptoms during pretreatment and posttreatment was performed and was assigned as a 5-point scale as follows: (1) worsening of symptoms; (2) no change in symptoms; (3) minor improvement of symptoms; (4) major improvement of symptoms; and (5) complete relief of symptoms. Patients were asked about the degree of satisfaction with their treatment and whether any side effects or complications occurred.

Complications included major and minor categories based on the Society of Interventional Radiology reporting standards (8).

## Results

### Patients and angiograms

Super-selective angiography demonstrated the internal maxillary and facial arteries of external carotid artery (*n* = 15), vertebral artery (*n* = 4), and occipital artery (*n* = 4). Of the total of 19 lesions, type IIIa + IIb (9 lesions; 47.5%), type IIIa + IIa (six lesions; 31.2%), and type IIIa +IV (3 lesions; 15.8%) were most common, followed by type IIIa (1 lesion; 5.3%) based on the classification by Yakes (14).

### Percutaneous suture of SDDOV

A total of 19 patients had RDDOV and received 42 percutaneous suture procedures (mean 2.2; range, 1–3). All patients had no immediate complications related to the suture of RDDOV. All patients had tolerable pain. No suture-associated hematoma or iatrogenic artery injury occurred.

### Embolization outcome

A total of 19 patients underwent 84 sessions of embolotherapy (mean 4.4; range, 2–8), which included 76 sessions using direct puncture, two using the transarterial method, and 6 using both. During the 84 ethanol embolization procedures performed on the 19 patients, the mean dose of ethanol used was 35.5 ml (range, 5–52 ml) for each treatment session. Notably, 16 patients had complete or >90% shunt reduction of the AVM, and three patients had >75% shunt reduction.

With regard to the clinical outcome, the control of swelling, bleeding, and pulsation was obtained after ethanol embolization. In all patients with Schobinger stage IV lesions at the initial presentation, the result of the post-embolization assessment showed that 4 patients had complete relief and 15 patients improved to stage I.

In 19 patients, dyspnea on exertion was the main reason for seeking intervention. The pulmonary catheter pressure (range, 42–58 mmHg) before embolization decreased (range, 28–37 mmHg) after embolization in all patients. The use of anti-failure medication before embolization in 5 patients was stopped after embolization. The symptom of dyspnea in all patients disappeared after embolization. All 19 (100%) patients presented with cardiac functional impairment before endovascular treatment. After treatment, the patients with NYHA II (*n* = 12), III (*n* = 5), and IV (*n* = 2) improved to NYHA I.

**Figure 3:**
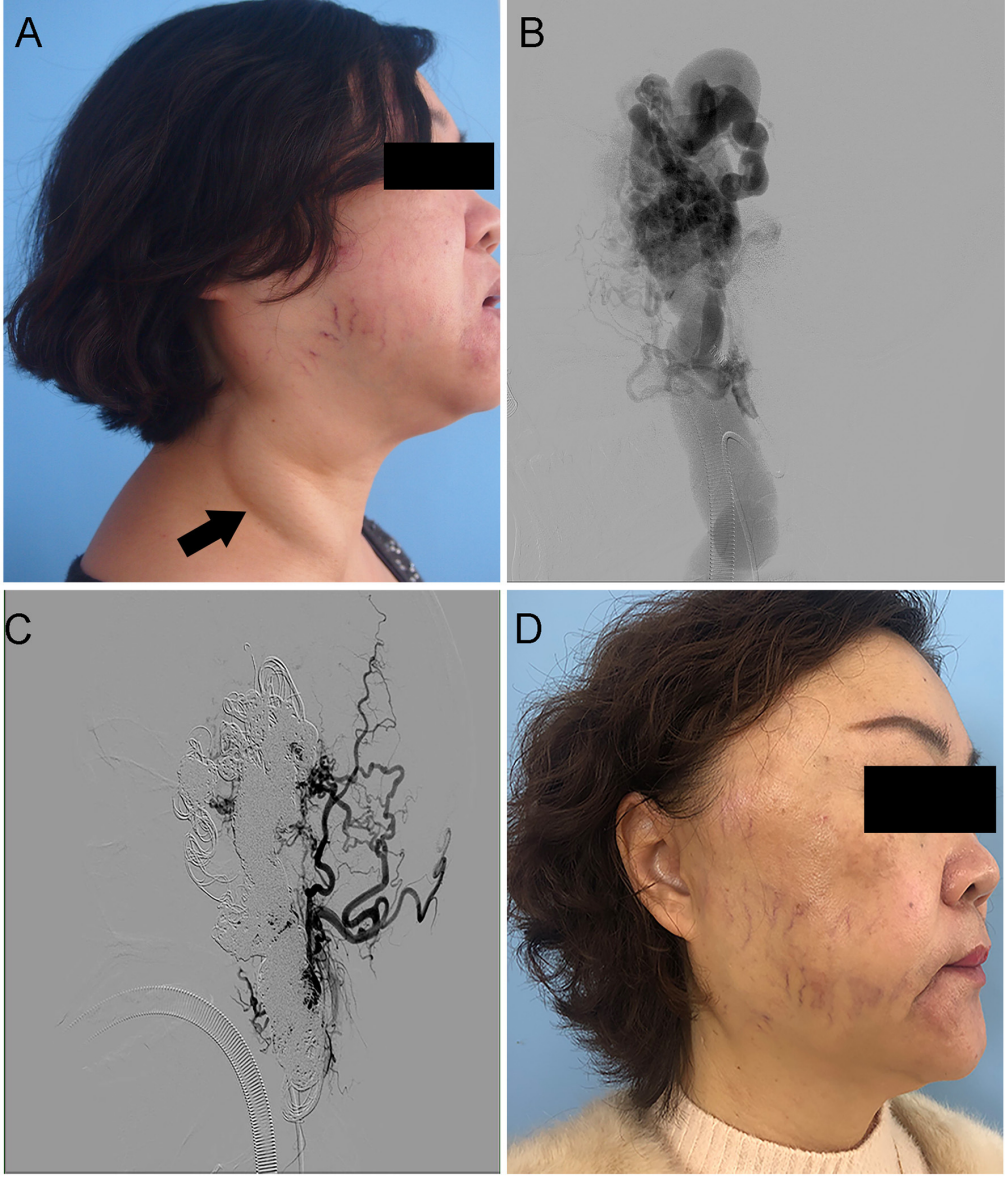
Case 6. An HOCF patient with right neck arteriovenous malformations (AVMs). **(A,B)** Preoperative photographs show giant right-neck AVMs with pulsatile masses. **(C,D)** Photographs taken 36 months after absolute ethanol and coil embolization embolotherapy of AVMs under the assistance of large neck reflux vein ligation show shrinkage of the neck AVMs without symptoms.

All 19 patients presented with impairment in daily life and cosmetic concerns. The impairment in daily life in 19 (100%) patients before endovascular treatment disappeared after treatment. Four patients (33.3%) had no cosmetic deformities after embolization (Figure 3), eight experienced major improvements in cosmetic deformities, and seven still had some minor improvements in cosmetic deformities. A worsening of cosmetic concerns did not occur. All patients reported satisfaction with the embolization treatment.

### Complications

Two patients developed the suture-associated infection, which was controlled by the topical antibiotics of erythromycin eye ointment. Transient hemoglobinuria disappeared around 5–6 h later in 17 observed patients with the treatment of continuous infusion of lactated Ringer solution intravenously. Renal impairment associated with hemoglobinuria was not present during the hospital stay. A total of five patients experienced blistering, which recovered spontaneously in 1 week. Two patients experienced transient nerve injuries of facial numbness that recovered spontaneously after 4 weeks. No major complications occurred. Notably, three patients had erosion and infection of coils through the skin surface, which required removal.

## Discussion

High-output cardiac failure caused by HNAVMs poses a problematic clinical scenario. The elimination of cardiac volume overload is the main goal of treatment for HNAVMs with HOCF. From the perspective of hemodynamics, the complete eradication of excessive arteriovenous shunts is an ideal option (3, 9). The treatment options included surgical excision, ligation of the feeding artery, and sclerotic embolization. Surgical resection is relatively risky due to the difficulty of controlling intraoperative bleeding, especially in the complicated head and neck areas. With the development of endovascular techniques and new embolic agents, embolization has gained popularity as the main treatment of choice to eradicate the underlying cause of the high-output state by closing arteriovenous fistula (AVF). The results demonstrated ethanol and coil embolotherapy for HNAVMs with HOCF using the percutaneous suture technique produced excellent angiographic and clinical results.

The available access to nidus is the transarterial approach, transvenous approach, and direct puncture approach. The choice of routine to nidus depends on the angioarchitecture. The previous report has revealed that for the large fistula, it is easy to get the draining vein through the transarterial approach and advance the microcatheter from the arterial part into the venous segment, coil from there and pull the catheter back. The transarterial approach was used in four procedures. In 15 patients who received percutaneous embolization, transarterial embolization seemed to be difficult owing to the failure of the transarterial approach, as indicated by numerous feeding arteries or previous transarterial embolization, indicating that the percutaneous approach is the mainstay approach. The direct puncture of AVMs with HOCF is advantageous for direct access to the nidus compared with the transarterial approach and transvenous approach. Based on this, a direct puncture approach was applied for the failure of the transarterial or transvenous approach.

Ethanol sclerotherapy often achieves symptomatic relief by reducing the blood flow and size of the lesion to an extent. The eradication of arteriovenous shunts requires complete damage to all three layers of the AVM vessel, allowing curative effects (3). Available agents for obliterating shunts, including polyvinyl alcohol, tissue adhesives, and coils, provide short-term symptomatic relief (10) and poor long-term results characterized by recanalization and neovascular recruitment (11–13). Ethanol has several advantages for managing complex AVMs, including destructive and curative properties (14). Its destructive effect is responsible for good clinical and radiologic results without recanalization and neovascular recruitment. Its destructive extent is associated with adequate contact with nidus (1).

High-output cardiac failure patients with HNAVMs often exhibit RDDOV, which plays a crucial role in AVM development and growth (4). Accumulating evidence demonstrates that the successful and effective management of RDDOV using coils optimizes AVM embolization with ethanol and yields outstanding angiographical and clinical outcomes (15, 16). In case of venous outflow significantly enlarged, previously reported treatment measures for RDDOV, including intertwining coils, an occlusion balloon, or a tourniquet (6, 17) are unsuitable in these cases. The management of significant RDDOV is challenging owing to difficulties associated with anchoring the coils in the candidate's vessel due to the significant high flow and the size of the RDDOV, which exceeds the effective diameter of the released coils available in our institution. In clinical practice, the available maximum diameter of detachable coils meets the criteria that the coils exceed 20–30% more than the inserted vessel diameter to prevent coil migration and distal embolization (17). Therefore, the high flow of RDDOV blood in AVMs with HOCF caused the failure of coils, increasing the risk of the migration of coils and the potential for catastrophic non-target embolism, including severe pulmonary or cardiovascular complications. From an anatomical point of view, RDDOV is located in the superficial region of the head and neck and is easy to identify and manage. To prevent the risk of coil migration, we introduced the RDDOV suture as an alternative method to provide a stable anchor for the deployed coil in the candidate's vessel. The percutaneous suture technique has the benefit of flow control, a decreased vessel diameter, and volume overload, providing a stable anchor for detachable coils and allowing for successful embolization in the RDDOV. In this study, there were no disastrous complications of coil migration due to the flow control by the RDDOV suture, indicating that RDDOV sutures are essential for coil embolization.

The percutaneous approach of the high-output fistula is associated with the risk of complications, including hematoma, or skin and subcutaneous tissue necrosis owing to ethanol leakage around the puncture site, which did not occur in this study. To avoid these complications, the treatment of the puncture site is crucial. Some measures were adopted about the sufficient manual compression of puncture after the withdrawal of the puncture needle, coil compact packing, and adequate dwelling time of ethanol before the withdrawal of the puncture needle. The decrease in the nidus volume by the suture technique potentially contributed to fewer embolization sessions and the total amount of used ethanol associated with complications. For AVM patients with HOCF, more attention should be paid to the toxicity of ethanol, which will further deteriorate cardiac function. Fortunately, the evidence from echocardiography supported that cardiac function improved. The potential explanation for the increased cardiac tolerance to ethanol toxicity is that coil embolization significantly obliterated the excessive shunts and decreased the volume overload. To detect the effect of injected ethanol on cardiac function, the placement of a pulmonary artery catheter is recommended (14, 18).

The expected results of embolization are relief of HOCF rather than healing, not complete eradication shunts, which is impossible to achieve in most cases. Staged embolization is recommended for the management of HOCF patients with AVMs to decrease the complications related to high doses of ethanol injected in a single session. This study has some limitations. First, it was a retrospective study. Second, a prospective study with a much bigger patient population is required to make a more accurate assessment. Finally, the number of patients in this study is small; therefore, it is difficult to have a proper statistic.

In this study, a surgical suture technique combination of percutaneous embolization using coils and ethanol achieved effective therapeutic outcomes in all patients with HOCF caused by HNAVMs.

## Data availability statement

The original contributions presented in the study are included in the article/supplementary material, further inquiries can be directed to the corresponding author.

## Ethics statement

Written informed consent was obtained from the individual(s) for the publication of any potentially identifiable images or data included in this article.

## Author contributions

All authors listed have made a substantial, direct, and intellectual contribution to the work and approved it for publication.

## Conflict of interest

The authors declare that the research was conducted in the absence of any commercial or financial relationships that could be construed as a potential conflict of interest.

## Publisher's note

All claims expressed in this article are solely those of the authors and do not necessarily represent those of their affiliated organizations, or those of the publisher, the editors and the reviewers. Any product that may be evaluated in this article, or claim that may be made by its manufacturer, is not guaranteed or endorsed by the publisher.
